# 淋巴结转移率与Ⅲa-N2非小细胞肺癌临床特征和生存相关性研究

**DOI:** 10.3779/j.issn.1009-3419.2019.11.04

**Published:** 2019-11-20

**Authors:** 善渊 张, 亮 王, 方亮 鲁, 宇权 裴, 跃 杨

**Affiliations:** 100142 北京，北京大学肿瘤医院暨北京肿瘤防治研究所胸外二科，恶性肿瘤发病机制及转化研究教育部重点实验室 Key Laboratory of Carcinogenesis and Translational Research (Ministry of Education), Department of Thoracic Surgery Ⅱ, Peking University Cancer Hospital and Institute, Beijing 100142, China

**Keywords:** 肺肿瘤, Ⅲa-N2, 淋巴结转移率, 生存, Lung neoplasms, Ⅲa-N2, Lymph node ratio, Prognosis

## Abstract

**背景与目的:**

淋巴结解剖位置是非小细胞肺癌N分期的基础，但Ⅲa-N2非小细胞肺癌生存差异显著，淋巴结转移率或与非小细胞肺癌预后有关，或可成为Ⅲa-N2非小细胞肺癌亚组评估指标。本研究探究淋巴结转移率与Ⅲa-N2非小细胞肺癌临床病理特征和生存的相关性。

**方法:**

纳入北京大学肿瘤医院胸外二科2006年1月-2016年12月期间直接手术的288例Ⅲa-N2非小细胞肺癌患者，卡方检验分析淋巴结转移率与临床病理特征相关性，*Cox*回归模型分析生存危险因素，*Kaplan*-*Meier*方法绘制生存曲线。

**结果:**

低淋巴结转移率139例，高淋巴结转移率149例，淋巴结转移率与腺癌（*χ*^2^=5.924, *P*=0.015）、最高组N2淋巴结转移（*χ*^2^=46.136, *P* < 0.001）、多个N2淋巴结转移（*χ*^2^=59.347, *P* < 0.001）、多站N2淋巴结转移（*χ*^2^=77.387, *P* < 0.001）及跳跃性N2淋巴结转移（*χ*^2^=61.524, *P* < 0.001）显著相关，与淋巴结清扫总数无关（*χ*^2^=0.537, *P*=0.464）。Cox回归模型多因素分析显示，腺癌（*P*=0.008）、多站N2淋巴结转移（*P*=0.025）和淋巴结转移率（*P*=0.001）是Ⅲa-N2非小细胞肺癌无病生存的独立危险因素，高淋巴结转移率组5年无病生存率为18.1%，低淋巴结转移率组5年无病生存率为44.1%。淋巴结转移率（*P* < 0.001）是Ⅲa-N2非小细胞肺癌总生存的独立危险因素，高淋巴结转移率组5年总体生存率为36.7%，低淋巴结转移率组5年总体生存率为64.1%。

**结论:**

淋巴结转移率与病理类型、跳跃性N2淋巴结转移、纵隔淋巴结转移个数、纵隔淋巴结转移站数、最高组N2淋巴结转移密切相关。淋巴结转移率是Ⅲa-N2非小细胞肺癌生存的独立危险因素，可更有效地预测Ⅲa-N2非小细胞肺癌亚组间的生存差异。

## Abstract (en)

**Background and Objective:**

Ⅲa-N2 non-small cell lung cancer was significant different in survival, although N stage of lung cancer based on anatomic location of metastasis lymph node. Lymph node ratio considered of prognostic factor might be the evaluation index for Ⅲa-N2 non-small cell lung cancer prognosis. Therefore, the aim of the study was to evaluate the correlation between lymph node ratio and clinicopathological features and prognosis of Ⅲa-N2 non-small cell lung cancer prognosis.

**Methods:**

A total of 288 cases of pathological Ⅲa-N2 non-small cell lung cancer were enrolled who received radical resection at the Department of Thoracic Surgery Ⅱ, Peking University Cancer Hospital from January 2006 to December 2016. The univariate analysis between clinicopathological variables and lymph node ratio used *Pearson's chi-squared* test. *Cox* regression was conducted to identify the independent prognosis factors for Ⅲa-N2 non-small cell lung cancer.

**Results:**

There were 139 cases in the lower lymph node ratio group, another 149 cases in the higher lymph node ratio group. Adenocarcinoma (*χ*^2^=5.924, *P*=0.015), highest mediastinal lymph node metastasis (*χ*^2^=46.136, *P* < 0.001), multiple-number N2 metastasis (*χ*^2^=59.347, *P* < 0.001), multiple-station N2 metastasis (*χ*^2^=77.387, *P* < 0.001) and skip N2 lymph node metastasis (*χ*^2^=61.524, *P* < 0.001) significantly impacted lymph node ratio. The total number of lymph node dissection was not correlated with the lymph node ratio (*χ*^2^=0.537, *P*=0.464). *Cox* regression analysis confirmed that adenocarcinoma (*P*=0.008), multiple-number N2 metastasis (*P*=0.025) and lymph node ratio (*P*=0.001) were the independent prognosis factors of disease free survival. The 5-year disease free survival was 18.1% in the higher lymph node ratio group, and 44.1% in the lower. Lymph node ratio was the independent prognosis factor of overall survival (*P* < 0.001). The 5-year overall survival was 36.7% in the higher lymph node ratio group, and 64.1% in the lower.

**Conclusion:**

Lymph node ratio was correlative with the pathology, highest mediastinal lymph node metastasis, multiple-number N2 metastasis, multiple-station N2 metastasis and skip N2 lymph node metastasis. Lymph node ratio was the independent prognosis factor for Ⅲa-N2 non-small cell lung cancer.

非小细胞肺癌淋巴结分期是评估生存的关键因素之一，Ⅲa-N2非小细胞肺癌5年生存率为35.0%-56.1%^[1-4]^。国际肺癌研究会（International Association for the Study of Lung Cancer, IASLC）尝试了以淋巴结解剖位置和淋巴结转移站数为基础的淋巴结分期方法，有利于区分不同亚组的生存差异^[1]^。淋巴结结转移率反映淋巴结转移数与淋巴结清扫总数比率，以往被文献报道为肺癌生存的独立危险因素^[5-7]^，但多数研究未阐述其与临床病理特征的相关性。因此，本研究拟回顾性研究Ⅲa-N2非小细胞肺癌临床病理特征与淋巴结转移率的相关性，以探究淋巴结转移率的生存预测价值。

## 1 资料与方法

### 1.1 一般资料

纳入北京大学肿瘤医院胸外二科2006年1月-2016年12月期间接受手术的Ⅲa-N2非小细胞肺癌病例。纳入标准：术后病理证实Ⅲa-N2非小细胞肺癌，已行根治性肺叶切除或联合肺叶切除，支气管残端阴性，清扫至少3站及以上纵隔淋巴结，清扫至少1站肺内淋巴结。排除接受术前新辅助治疗以及多发病灶无法证实多原发的病例。最后，共288例病理Ⅲa-N2非小细胞肺癌被纳入研究，其中男性141例，女性147例，中位年龄61岁。病理类型包括腺癌223例，非腺癌65例，T1期78例，T2a期158例，T2b期52例，中位肿瘤大小3.0 cm，中位淋巴结清扫数20个。

### 1.2 研究方法

本研究采用IASLC推荐的第八版TNM分期，以IASLC淋巴结图谱为依据定义N2站淋巴结^[8]^。记录N2淋巴结清扫站数及个数、N2淋巴结转移站数及个数，记录N1淋巴结清扫站数及个数、N1淋巴结转移站数及个数。最高组N2淋巴结转移是指清扫的最高解剖位置的纵隔淋巴结发生转移，右侧纵隔淋巴结排序为第2R、3A、4R、7组，左侧纵隔淋巴结排序为第4L、5、6、7组。跳跃性N2淋巴结转移是指N1站淋巴结阴性而N2站纵隔淋巴结发生癌转移。淋巴结转移率（lymph node ratio, LNR）是指淋巴结转移个数与淋巴结清扫总数之比，采用*Cox*风险回归模型确定淋巴结转移率最佳截点^[9]^，因此可分为低淋巴结转移率组（LNR≤0.2）和高淋巴结转移率组（LNR > 0.2）。生存随访方式包括门诊随访及电话随访，中位随访时间38.7个月。

### 1.3 统计学方法

采用SPSS 22.0统计软件数据分析，分类计数四格表采用卡方检验，*Cox*回归模型分析生存独立危险因素，生存曲线图采用GraphPad Prism 6.0软件绘制，*Log*-*rank*检验分析组间生存率差异。所有检验采用双侧检验，*P* < 0.05视为统计学上有显著差异。

## 2 结果

### 2.1 临床病理特征相关性分析

单因素分析淋巴结转移率与临床病理特征相关性，结果见表 1。纳入研究的病例中，低淋巴结转移率组有139例，高淋巴结转移率组149例。卡方检验显示，淋巴结转移率与性别、年龄、肿瘤大小、T分期、胸膜侵犯、脉管癌栓无关，而腺癌更容易发生淋巴结转移（*χ*^2^=5.924, *P*=0.015）。分析显示，清扫16个以上淋巴结与淋巴结转移率高低无相关性（*χ*^2^=0.537, *P*=0.464），最高组N2淋巴结转移与高淋巴结转移率具有显著相关性，高淋巴结转移率组发生最高组N2淋巴结转移比例更高（*χ*^2^=46.136, *P* < 0.001），同时也更容易发生多个N2淋巴结转移（*χ*^2^=59.347, *P* < 0.001）和多站N2淋巴结转移（*χ*^2^=77.387, *P* < 0.001），跳跃性N2淋巴结转移者淋巴结转移率低（*χ*^2^=61.524, *P* < 0.001）。

**1:** 临床病理特征与淋巴结转移率相关性 Correlation between clinicopathological features and lymph node ratio

Characteristic	*N*	LNR≤0.2 [*n* (%)]	LNR > 0.2 [*n* (%)]	*χ*^2^	*P*
Age (yr)				0.219	0.640
≤60	143	71（49.7）	72（50.3）		
> 60	145	68（46.9）	77（53.1）		
Gender				0.050	0.823
Male	141	69（48.9）	72（51.1）		
Female	147	70（47.6）	77（52.4）		
Histology				5.924	0.015
Adenocarcinoma	223	99（44.4）	124（55.6）		
Others	65	40（61.5）	25（38.5）		
Tumor size				0.157	0.692
≤3 cm	154	76（49.4）	78（50.6）		
> 3 cm	134	63（47.0）	71（53.0）		
T stage				0.029	0.864
T1	78	37（47.4）	41（52.6）		
T2	210	102（48.6）	108（51.4）		
VPI				0.812	0.368
Yes	155	71（45.8）	84（54.2）		
No	133	68（51.1）	65（48.9）		
LVI				3.478	0.062
Yes	107	44（41.1）	63（58.9）		
No	181	95（52.5）	86（47.5）		
No. of LN resected				0.305	0.581
< 16	62	28（45.2）	34（54.8）		
≥16	226	111（49.1）	115（50.9）		
Highest MLN station positive				46.163	< 0.001
Yes	132	35（26.5）	97（73.5）		
No	156	104（66.7）	52（33.3）		
No. of positive N2				59.347	< 0.001
Single	94	76（80.9）	18（19.1）		
Multiple	194	63（32.5）	131（67.5）		
Positive N2 stations				77.387	< 0.001
Single	155	112（72.3）	43（27.7）		
Multiple	133	27（20.3）	106（79.7）		
Skip N2 metastasis				61.524	< 0.001
Yes	81	69（85.2）	12（14.8）		
No	207	70（33.8）	137（66.2）		
VPI: Visceral pleural invasion; LVI: lymphvascular invasion; MLN: mediastinal lymph node.					

### 2.2 单因素分析非小细胞肺癌生存危险因素

采用*Cox*回归模型单因素分析非小细胞肺癌生存危险因素，结果见表 2。无病生存危险因素分析显示，性别、年龄、肿瘤大小、T分期、脉管癌栓以及清扫淋巴结总数与无病生存无显著相关性。腺癌（*P*=0.002）、胸膜侵犯（*P*=0.022）、最高组淋巴结转移（*P* < 0.001）、多个N2淋巴结转移（*P* < 0.001）、多站N2淋巴结转移（*P* < 0.001）、非跳跃性N2淋巴结转移（*P*=0.009）以及高淋巴结转移率（*P* < 0.001）具有更低的无病生存率。

**2:** *Cox*回归单因素分析生存危险因素 Univariable analysis of survival risk factors with *Cox* regression

Characteristic	DFS					OS			
	HR	95%CI		*P*		HR	95%CI		*P*
		Lower	Upper				Lower	Upper	
Age (> 60 yr *vs* ≤60 yr)	1.002	0.750	1.337	0.991		1.146	0.815	1.613	0.433
Gender (Female *vs* Male)	1.069	0.800	1.427	0.653		0.856	0.609	1.203	0.370
Histology (Adenocarcinoma *vs* Others)	1.796	1.235	2.613	0.002		0.826	0.549	1.243	0.359
Tumor size (> 3 cm *vs* ≤3 cm)	1.113	0.834	1.485	0.468		1.331	0.947	1.871	0.099
T stage (T2 *vs* T1)	1.177	0.849	1.632	0.329		1.145	0.775	1.690	0.496
VPI (Yes *vs* No)	1.409	1.050	1.889	0.022		1.136	0.807	1.600	0.464
LVI (Yes *vs* No)	1.228	0.915	1.650	0.172		1.195	0.842	1.696	0.319
No. of LN resected (≥16 *vs* < 16)	1.007	0.707	1.435	0.969		0.904	0.602	1.360	0.629
Highest MLN station positive (Yes *vs* No)	1.723	1.288	2.305	< 0.001		1.548	1.099	2.179	0.012
No. of positive N2 (Multiple *vs* Single)	1.857	1.329	2.595	< 0.001		1.647	1.111	2.442	0.013
Positive N2 stations (Multiple *vs* Single)	1.986	1.482	2.659	< 0.001		1.803	1.279	2.540	0.001
Skip N2 (No *vs* Yes)	1.589	1.125	2.244	0.009		1.518	1.010	2.283	0.045
LNR (> 0.2 *vs* ≤0.2)	2.353	1.737	3.187	< 0.001		2.352	1.641	3.372	< 0.001
LNR: lymph node ratio; DFS: disease free survival; OS: overall survival.									

总生存危险因素分析显示，性别、年龄、病理类型、肿瘤大小、T分期、脉管癌栓、胸膜侵犯、清扫淋巴结总数与总生存无关。最高组N2淋巴结转移（*P*=0.012）、多个N2淋巴结转移（*P*=0.013）、多站N2淋巴结转移（*P*=0.001）、非跳跃性N2淋巴结转移（*P*=0.009）以及高淋巴结转移率（*P* < 0.001）总生存期更短。

### 2.3 多因素分析非小细胞肺癌生存危险因素

采用*Cox*回归模型多因素分析生存危险因素，结果见表 3。无病生存多因素分析显示，腺癌（*P*=0.008）、多站N2淋巴结转移（*P*=0.025）和淋巴结转移率（*P*=0.001）是Ⅲa-N2非小细胞肺癌无病生存的独立危险因素。生存曲线分析显示，高淋巴结转移率组5年无病生存率为18.1%，而低淋巴结转移率组5年无病生存率为44.1%，结果见图 1A。

**3:** *Cox*回归多因素分析生存危险因素 Multivariable analysis of survival risk factors with *Cox* regression

Characteristic	DFS					OS			
	HR	95%CI		*P*		HR	95%CI		*P*
		Lower	Upper				Lower	Upper	
Histology (Adenocarcinoma *vs* Others)	1.673	1.144	2.447	0.008					
VPI (Yes *vs* No)	1.227	0.913	1.648	0.175					
Highest MLN station positive (Yes *vs* No)	1.121	0.785	1.601	0.531		1.009	0.668	1.524	0.967
No. of positive N2 (Multiple *vs* Single)	1.097	0.697	1.728	0.688		0.953	0.561	1.619	0.858
Positive N2 stations (Multiple *vs* Single)	1.480	1.051	2.083	0.025		1.256	0.848	1.860	0.255
Skip N2 (No *vs* Yes)	1.012	0.688	1.490	0.950		1.022	0.651	1.605	0.925
LNR (> 0.2 *vs* ≤0.2)	1.823	1.276	2.603	0.001		2.352	1.641	3.372	< 0.001

**1:**
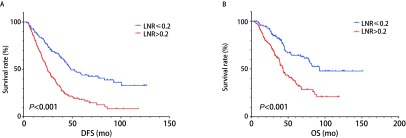
淋巴结转移率生存曲线图。A：淋巴结转移率无病生存曲线；B：淋巴结转移率总生存曲线。 The survival curve of lymph node ratio (LNR). A: Disease free survival with different LNR; B: Overall survival with different LNR.

总生存多因素分析显示，淋巴结转移率（*P* < 0.001）是Ⅲa-N2非小细胞肺癌总生存的独立危险因素，高淋巴结转移率死亡风险增加2.352倍。生存曲线分析显示，高淋巴结转移率组5年总体生存率为36.7%，而低淋巴结转移率组5年总体生存率为64.1%，结果见图 1B。

## 3 讨论

外科手术仍是Ⅲa-N2非小细胞肺癌治疗的主要手段之一，一方面可能达到肿瘤完全性切除，另一方面可获取准确的肿瘤分期，尤其是N分期，详尽的病理分期对治疗策略制定和预后评估至关重要^[10]^。美国国家综合癌症网络（National Comprehensive Cancer Network, NCCN）推荐的肺癌手术完全切除标准被广泛应用，Raymond等^[11]^曾研究发现此质量控制标准可提高非小细胞肺癌生存获益，尤其是淋巴结切除质量。对于Ⅰ期-Ⅲa期非小细胞肺癌，研究推荐清扫16个及以上淋巴结可获得更准确的淋巴结分期^[12]^。有研究^[13]^对比不同淋巴结清扫质量控制标准发现，清扫至少3站纵隔淋巴结可更准确地反映非小细胞肺癌N1与N2的生存差异。本研究队列纳入清扫3站及以上纵隔淋巴结Ⅲa非小细胞肺癌病例，中位淋巴结清扫数20个，清扫16个及以上淋巴结占78.5%，5年总生存率为49.9%，高于日韩及欧美报道同期生存数据^[14-16]^。

IASLC推荐的第八版肿瘤-淋巴结-转移（tumor-node-metastasis, TNM）分期仍是以淋巴结解剖位置作为非小细胞肺癌N分期的主要依据，但同一分期亚组间生存差异明显，有文献报道提出淋巴结转移率、淋巴结转移个数、淋巴结转移站数等可补充甚至替代现有的N分期^[6, 16-18]^。淋巴结转移率是淋巴结转移数与淋巴结清扫总数的比值，其可靠性需要依靠规范的淋巴结清扫和病理评估^[19]^。淋巴结转移率作为乳腺癌、结肠癌预测指标最早被研究，Wisnivesky等^[20]^最先研究发现高淋巴结转移率是老年N1非小细胞肺癌高危复发风险因素。Tamura等^[5]^研究发现淋巴结转移率 > 22%时，N2非小细胞肺癌总生存风险增加1.725倍。本研究多因素分析发现，淋巴结转移率是Ⅲa-N2非小细胞肺癌生存评估的独立危险因素。高淋巴结转移率组肿瘤复发风险增加1.823倍，其5年生存率仅为18.1%，而低淋巴结转移率组5年生存率仅为44.1%。高淋巴结转移率组的肿瘤死亡风险增加2.352倍，5年生存率为36.7%，而低淋巴结转移率组为64.1%。正如相关文献报道，不仅低淋巴结转移率与非小细胞肺癌良好预后相关，更多的淋巴结清扫数也可提高生存获益^[21]^。

本研究分析淋巴结转移率相关因素发现，淋巴结转移率与淋巴结清扫总数无相关性，规范的淋巴结清扫质量控制可更准确的分期，淋巴结清扫数对同一分期的预后差异的影响降低。同时，高淋巴结转移率与腺癌、跳跃性淋巴结转移、最高组N2淋巴结阳性、多个纵隔淋巴结转移及多站纵隔淋巴转移相关。淋巴结转移与病理类型有关，腺癌更容易发生淋巴结转移，尤其是微乳头及实性成分为主的腺癌^[22, 23]^。Riquet等^[16]^发现跳跃性N2转移是非小细胞肺癌生存的有利因素，Asamura等^[24]^研究结果建议利用淋巴结解剖位置、淋巴结转移站数及跳跃转移评估非小细胞肺癌预后。但是，跳跃性N2淋巴结转移与非小细胞肺癌预后相关性仍有争议，Tamura等^[5]^研究发现跳跃N2转移与N2非小细胞肺癌生存无相关性。我们研究通过*Cox*回归单因素分析发现，Ⅲa-N2非小细胞肺癌跳跃N2转移预后较好，但多因素分析显示跳跃性N2转移不是独立预测因素。

同时，单因素分析结果显示，Ⅲa-N2非小细胞肺癌多个纵隔淋巴结转移和多站纵隔淋巴结转移与生存具有差异性。Fan等^[18]^研究建议N分期联合淋巴结个数预测Ⅲ期非小细胞肺癌生存。Cho等^[4]^发现多站纵隔淋巴结转移N2非小细胞肺癌5年生存率为36.4%，而单站转移5年生存率为66.6%。纵隔最高组淋巴结阳性提示手术未到达完全性切除，本研究队列中有45.8%的Ⅲa-N2非小细胞肺癌纵隔最高组淋巴结阳性，其复发及转移风险也相应增高。Zheng等^[25]^研究报道病理N2非小细胞肺癌最高组N2淋巴结阳性为44.8%，最高组纵隔淋巴结阳性生存较差，Gagliasso等^[26]^研究也证实了相同观点。

总的来说，我们通过回顾性研究淋巴结转移率与Ⅲa-N2非小细胞肺癌临床病理特征及生存的关系发现，淋巴结转移率是Ⅲa-N2非小细胞肺癌生存的独立危险因素，可更有效预测Ⅲa-N2非小细胞肺癌不同亚组间的生存差异。淋巴结转移率与病理类型、跳跃性淋巴结转移、纵隔淋巴结转移个数、纵隔淋巴结转移站数、最高组N2淋巴结转移密切相关。但本研究是单中心回顾性小样本研究，需要更大样本量的多中心研究提供更多循证医学证据。
